# Poor mental health and its impact on academic outcomes in university students before and during the COVID-19 pandemic: analysis of routine service data

**DOI:** 10.1192/bjo.2024.868

**Published:** 2025-03-11

**Authors:** Brian C. F. Ching, Jane S. Hahn, Sarah Corrie, Rhiannon Thomas, Anca Alba

**Affiliations:** Department of Child and Adolescent Psychiatry, Institute of Psychiatry, Psychology & Neuroscience, King’s College London, UK; Division of Psychiatry, University College London, UK; School of Social Sciences and Humanities, University of Suffolk, UK; Counselling and Mental Health Support Service, King’s College London, UK

**Keywords:** COVID-19, explanatory factors, student mental health, university students

## Abstract

**Background:**

There is an urgent need to understand explanatory factors of poor mental health before (pre-) and during (peri-) the COVID-19 pandemic in university students, especially those from underrepresented and minority groups.

**Aims:**

To examine potential differences and explanatory factors for psychological distress, clinical risk and impact of problems on academic outcomes pre- and peri-pandemic in university students.

**Method:**

A repeated cross-sectional design was used with routine data between August 2018 and July 2022 at the registration stage from a student counselling and mental health service at a UK university. Wilcoxon signed-rank tests were used to examine pre- and peri-pandemic differences in outcomes. Unadjusted and adjusted linear regression models were conducted to assess potential explanatory factors for poor outcomes.

**Results:**

A total of 9517 university students had completed sociodemographic and outcome data and were included in analysis. Psychological distress and impact of problems on academic outcomes were not significantly different between pre- and peri-pandemic groups. Clinical risk was significantly higher in the pre-pandemic than peri-pandemic group. Potential explanatory factors for poorer outcomes included being younger, female or non-binary/genderqueer, sexual minority, from a minority ethnic group, having home fee status and having a disability registration.

**Conclusions:**

Poor student mental health profiles and related explanatory factors may not have changed drastically between pre- and peri-pandemic. Longitudinal methods and intersectional approaches should be used in future research. Further understanding of how universities and student mental health services can most efficiently and effectively support the mental health of university students is crucially warranted.

The coronavirus (COVID-19) pandemic introduced unprecedented changes for students, such as exam cancellations, escalation of digital learning and uncertainty over their futures. University students had to face additional challenges, including virtual campuses, socially distanced lectures and the closing of student spaces. The impact of the pandemic may have exacerbated pre-existing concerns of increases in prevalence of mental health problems in university students.^1^

During the pandemic, several surveys reported high levels of psychological distress, anxiety, depression and stress.^2–6^ A cross-sectional survey of 895 university students in the UK found that approximately 40% met the threshold for moderate to severe anxiety and depression in summer 2020.^2^ A longitudinal survey of 254 university students, also in the UK, found a significant increase in depressive symptoms and decrease in well-being during the April–May 2020 lockdown compared with pre-pandemic periods.^3^ Moreover, increased mental health problems during the pandemic may be associated with academic outcomes; a cross-sectional survey of 5021 students across four universities in Germany found high levels of depression and approximately half of university students were struggling with workload, felt overwhelmed with increases in workload and were worried about not being able to complete the academic year.^7^ However, most studies do not have both pre- and peri-pandemic data, peri-pandemic data beyond late 2020 and/or large samples, which limits the comparison of student mental health profiles.

## Research priorities

Research priorities informed by university students’ experiences highlight an urgent need to understand the explanatory factors of poor mental health, especially experiences of students from minority groups.^8^ Cross-sectional surveys have found potential risk factors for poor mental health and its impact on academic outcomes in university students, such as being female or non-binary,^2,5,6,9,10^ of younger age,^2,10^ from a minority ethnic group^10^ and having a disability.^11–13^ Additionally, surveys using samples of lesbian, gay, bisexual and transgender (LGBT) university students found relatively high psychological distress, anxiety and depression during the pandemic.^14,15^ Understanding explanatory factors for poor student mental health, especially during the pandemic, is urgently needed to inform service provision in university mental health services. However, most studies assess characteristics only broadly (e.g. Asian or LGBT) or not at all (e.g. disability and student fee status are not routinely collected). These measurement limitations prevent finer-grained analysis of potential mental health differences across student groups to understand what drives these changes.

This study aims to use routine university student service entry data to understand psychological distress, clinical risk and impact on academic outcomes. Specifically, we aim to investigate: (a) whether psychological distress, clinical risk and impact of problems on academic outcomes at service entry differed before and during the pandemic; and (b) whether psychological distress, clinical risk and impact of problems on academic outcomes at service entry were associated with sociodemographic factors (age, gender, ethnicity, sexual orientation, fee status and disability status) before and during the pandemic.

## Method

### Study design

We used a repeated cross-sectional design. Data were collected from students (*n* = 10 851) who attended a student counselling and mental health support (CMHS) service in a university in London, UK, across four academic years (August 2018 to July 2019, August 2019 to July 2020, August 2020 to July 2021, and August 2021 to July 2022). The university is one of the largest in London, with a diverse community of over 47 000 undergraduate and postgraduate students from 160 countries, and a higher representation of minority ethnic and gender groups compared with the UK population. Data were collected as routine measures completed by students on registration with the service to inform the initial assessment process. Students with complete sociodemographic and outcome data were included in the analysis sample. Approval for secondary data analysis of routine service data was granted by King’s College London University Research Data Storage and students provided consent to the service privacy policy, which permits data collection and reporting for audit and service improvement purposes.

### Sample

Students completed an online self-referred form to register with the CMHS service. To enter the service, students had to meet the following inclusion criteria: (a) enrolled at the university for a degree programme, (b) registered with a general practitioner in the UK, (c) and not have already registered with the service in the past 3 months. Once registered, students were offered an initial assessment and brief psychological intervention for mild to moderate mental health problems by qualified counsellors (e.g. counselling and clinical psychologists, psychotherapists and mental health advisors). Students with complete sociodemographic and routine mental health measures data were included in analysis.

### Measures

The online registration form that students complete at service entry includes measures on sociodemographic information, mental health and perceived impact of problems on academic outcomes. Students were also given these same questions at end of treatment. However, only measures assessed at service entry are used in this paper, as we were only interested in mental health profiles in students when presenting to the service.

#### Pre- and peri-pandemic

We present four academic years of data (August 2018–July 2022), which covered three national lockdowns, implementation of various social restrictions and changes in service delivery. In the UK, three national lockdowns occurred: March–June 2020, November–December 2020 and January–March 2021. At the start of the pandemic, university teaching and CMHS service delivery were moved online, and students were advised to return home. Service delivery then transitioned to a hybrid mode in the 2020–2021 academic year and moved fully back to in-person in 2021–2022. Academic years at the student’s entry to the service were combined into pre-pandemic (August 2018 to July 2019 and August 2019 to 17 March 2020) and peri-pandemic (18 March 2020 to July 2022) groups.

#### Sociodemographic variables

Self-reported sociodemographic information was assessed, including age, gender, ethnicity, sexual orientation, fee status and disability status. Age was treated as a continuous and categorical variable (16–24, 25–34 and ≥34 years). Gender was a categorical variable with male, female and other gender (non-binary/genderqueer) options. Ethnicity was categorised into Black (African, Caribbean and any other Black background), South Asian (Bangladeshi, Indian and Pakistani), Chinese, Other Asian (any other Asian background), White British, Other White (White Irish and any other White background), Mixed (White and Asian, White and Black African, White and Black Caribbean, and any other Mixed background) and Other ethnic background options. Although it was possible to have even more granular ethnic categories, we used these categories to ensure that there were enough respondents in each group. Sexual orientation was a categorical variable with heterosexual, bisexual, gay/lesbian and not sure/queer options. Fee status was a categorical variable with home, European Union (EU) and overseas student options. Disability was a categorical variable with disability registration and no disability registration options.

#### Outcomes

Psychological distress and clinical risk were assessed using the Clinical Outcomes in Routine Evaluation (CORE-OM) scale.^16^ The 34-item self-report measure assesses psychological distress in the past week across four domains: subjective well-being, mental health problems and their symptoms, life functioning and risk/harm. Scores are presented as a total raw score (0–40), where higher scores represent worse psychological distress.^17^ A total clinical risk score was calculated by totalling the risk/harm domain, presented as total scores of 0–4.^18^ The clinical cut-off for the CORE-OM total and risk scores are 10 and 1 respectively.

Impact of problems on academic outcomes was assessed using the Counselling Impact on Academic Outcomes (CIAO) scale.^19^ The 9-item self-report measure assesses the perceived impacts of their problems on academic outcomes (questions 1–3) and of counselling on their academic outcomes (questions 4–9). In this study, we only use scores from questions 1–3, as only these were asked at service entry. The perceived impact of their problems on academic outcomes are presented as a total raw score (3–15), where higher scores indicate that problems more frequently had a negative impact on their academic outcomes, such as thoughts of leaving their course, ability to study and overall student experience.

### Statistical analysis

Analyses were done using Stata 17 for Windows. For descriptive analyses, we summarised our sample by sociodemographic variables, both for complete and missing data.

For research question 1 (whether psychological distress, clinical risk and impact of problems on academic outcomes at service entry differed before and during the pandemic), we first tested the normal distribution of the outcomes. As the data were not normally distributed, Wilcoxon signed-rank tests were used to examine mean differences between the two groups of students, with the pandemic as the independent categorical variable (pre- and peri-pandemic) and psychological distress (CORE-OM total score), clinical risk (CORE-OM risk score) and impact of problems on academic outcomes (CIAO total score) as the dependent variables.

For research question 2 (potential explanatory factors for psychological distress, clinical risk and impact of problems on academic outcomes), we ran separate univariable linear regression models to assess the unadjusted association between sociodemographic variables (age, gender, ethnicity, sexual orientation, fee status and disability status) and psychological distress, clinical risk and impact of problems on academic outcomes in the whole cohort. We then adjusted the model for all other sociodemographic variables.

We performed sensitivity analyses too. For research question 1, we investigated whether the differences in outcomes between pre- and peri-pandemic were explained by academic years (August 2018 to July 2019, August 2019 to July 2020, August 2020 to July 2021, and August 2021 to July 2022) using one-way analysis of variance (ANOVA) and *post hoc* Tukey tests. For research question 2, we investigated whether associations between sociodemographic variables and outcomes differed between pandemic time points by running regression models with the separate pre- and peri-pandemic groups. Additionally, we imputed sociodemographic variables and outcomes in 50 data-sets and reran the separate unadjusted and adjusted models. To impute missing data using multiple imputation by chained equations, we used all sample characteristics, outcomes and pandemic time point (pre- and peri-pandemic) as an auxiliary variable.

## Results

### Participant characteristics

Of the respondents included in the analysis (*n* = 9517), 4522 presented to the service pre-pandemic and 4995 peri-pandemic (Table 1). Over 76% of students were 16–24 years old and 77.5% were female. Most students identified as heterosexual (73.9%). The largest ethnic groups in the sample were White British (29.7%) and Other White (21.8%). The majority of students were classified as home students (63%) and few students were registered as having a disability (12.6%). Students who presented to the CMHS service between the pre- and peri-pandemic time points shared differences across all sample characteristics. The sample’s outcomes were clinically significant, with the following mean (s.d.) scores: 19.60 (5.81) on the CORE-OM total score; 2.46 (3.27) on the CORE-OM risk score; and 6.73 (2.87) on the CIAO total score. The 538 students who did not have all outcome variables available were excluded from the analysis (Supplementary Table 1, available at https://doi.org/10.1192/bjo.2024.868).

**Table 1:** Characteristics of students with complete data for all sociodemographic and outcome variables

Characteristics	Whole cohort (*n* = 9517)	Pre-pandemic (*n* = 4522)	Peri-pandemic (*n* = 4995)	Comparison *z* or χ^2^
Age, years: mean (s.d.)	22.80 (4.61)	22.69 (4.66)	22.89 (4.57)	*z* = −3.68, *P* = 0.002 χ^2^ = 9.00, *P* = 0.011
16–24	7290 (76.60)	3520 (77.86)	3769 (75.36)	
25–34	1962 (20.62)	873 (19.31)	1089 (21.80)	
≥35	265 (2.78)	128 (2.83)	137 (2.74)	
Gender, *n* (%)				χ^2^ = 16.60, *P* = 0.000
Male	2051 (21.55)	990 (21.89)	1061 (21.24)	
Female	7378 (77.52)	3509 (77.60)	3869 (77.46)	
Other	88 (0.92)	23 (0.51)	65 (1.30)	
Sexual orientation, *n* (%)				χ^2^ = 27.98, *P* = 0.000
Heterosexual	7029 (73.86)	3442 (76.12)	3587 (71.81)	
Bisexual	1140 (11.98)	482 (10.66)	658 (13.17)	
Gay/lesbian	454 (4.77)	219 (4.84)	235 (4.70)	
Not sure/queer	894 (9.39)	379 (8.38)	515 (10.31)	
Ethnicity, *n* (%)				χ^2^ = 29.88, *P* = 0.000
Black	567 (5.96)	245 (5.42)	322 (6.45)	
South Asian	1397 (14.68)	623 (13.78)	774 (15.50)	
Chinese	792 (8.32)	338 (7.47)	454 (9.09)	
Other Asian	608 (6.39)	276 (6.10)	332 (6.65)	
White British	2829 (29.73)	1408 (31.14)	1421 (28.45)	
Other White	2076 (21.81)	1044 (23.09)	1032 (20.66)	
Mixed	822 (8.64)	390 (8.62)	432 (8.65)	
Other	426 (4.48)	198 (4.38)	228 (4.56)	
Fee status, *n* (%)				χ^2^ = 16.14, *P* = 0.000
Home	5994 (62.98)	2776 (61.39)	3218 (64.42)	
European Union	1507 (15.83)	785 (17.36)	722 (14.45)	
Overseas	2016 (21.18)	961 (21.25)	1055 (21.12)	
Disability registration, *n* (%)				χ^2^ = 9.12, *P* = 0.003
Yes	1202 (12.63)	620 (13.71)	582 (11.65)	
No	8315 (87.37)	3902 (86.29)	4413 (88.35)	

### Missing data

Of the whole cohort (*n* = 10 055), 9517 had complete sociodemographic and outcome data and 538 were missing at least one sociodemographic or outcome data (Supplementary Table 1). Within the pre-pandemic group (*n* = 4666), 4522 had complete sociodemographic and outcome data and 144 had some missing sociodemographic and outcome data. Within the peri-pandemic group (*n* = 5389), 4995 had complete sociodemographic and outcome data and 394 had some missing sociodemographic and outcome data.

A similar proportion of the age, sexual orientation, fee status and disability groups had missing measurements for sociodemographic and outcome data across the pre- and peri-pandemic time points. Male students had a similar proportion of missing measurements for sociodemographic and outcome data compared with female students across the pre- and peri-pandemic time points, but non-binary/genderqueer students had a higher proportion of missing measurements for sociodemographic and outcome data at the pre-pandemic time point. Black, South Asian, Other Asian, White British, Other White, Mixed and Other ethnic groups had a similar proportion of missing measurements for sociodemographic and outcome data across the pre- and peri-pandemic time points, except Chinese students had a higher proportion of missing data at the peri-pandemic time point.

### Differences in outcomes between pre- and peri-pandemic participants

As presented in Table 2 (*n* = 9517), students who entered the service pre-pandemic did not have significantly different mean psychological distress (*z* = 1.416, Cohen’s *d* = 0.03, 95% CI −0.007 to 0.074) or mean impact of problems on academic outcomes (*z* = 1.329, Cohen’s *d* = 0.03, 95% CI −0.008 to 0.073) compared with students who entered the service during the pandemic. Mean clinical risk was significantly higher in students who entered services pre-pandemic compared with peri-pandemic, albeit with very small effect size (*z* = 2.863, Cohen’s *d* = 0.04, 95% CI 0.003–0.083).

**Table 2:** CORE-OM and CIAO scores for the pre- and peri-pandemic samples (*n* = 9517)

Scales	Pre-pandemic, mean (s.d.)	Peri-pandemic, mean (s.d.)	Wilcoxon signed-rank test	Cohen’s *d*	95% CI
CORE-OM total	19.69 (5.82)	19.49 (5.79)	*z* = 1.416, *P* = 0.157	0.03	−0.007 to 0.074
CORE-OM risk	2.54 (3.31)	2.40 (3.24	*z* = 2.863, *P* = 0.004	0.04	0.003 to 0.083
CIAO total	6.85 (2.79)	6.76 (2.81)	*z* = 1.329, *P* = 0.184	0.03	−0.008 to 0.073

CORE-OM, Clinical Outcomes in Routine Evaluation; CIAO, Counselling Impact on Academic Outcomes.

### Potential explanatory factors

Unadjusted and adjusted linear regression analyses (*n* = 9517) are presented in Tables 3, 4 and 5.

**Table 3:** Linear regression analysis on the association between potential explanatory factors and CORE-OM total score (*n* = 9517)

	Unadjusted		Fully adjusted	
Fixed effects	β or mean difference (95% CI)	*P*	β or mean difference (95% CI)	*P*
Age	−0.072 (−0.097 to −0.047)	<0.001	−0.067 (−0.093 to −0.042)	<0.001
Gender				
Male	1		1	
Female	0.804 (0.521 to 1.088)	<0.001	0.746 (0.463 to 1.028)	<0.001
Other	2.097 (0.861 to 3.333)	0.001	1.488 (0.246 to 2.731)	0.019
Sexual orientation				
Heterosexual	1		1	
Bisexual	0.939 (0.577 to 1.301)	<0.001	0.830 (0.466 to 1.194)	<0.001
Gay/lesbian	−0.375 (−0.924 to 0.175)	0.182	−0.099 (−0.654 to 0.457)	0.727
Not sure/queer	0.907 (−0.504 to 1.310)	<0.001	0.789 (0.386 to 1.192)	<0.001
Ethnicity				
Black	1.280 (.761 to 1.800)	<0.001	1.350 (.831 to 1.869)	<0.001
South Asian	1.475 (1.106 to 1.844)	<0.001	1.715 (1.336 to 2.095)	<0.001
Chinese	1.148 (.694 to 1.602)	<0.001	1.740 (1.215 to 2.264)	<0.001
Other Asian	1.719 (1.215 to 2.224)	<0.001	2.062 (1.540 to 2.585)	<0.001
White British	1		1	
Other White	−0.224 (−0.550 to 0.102)	0.178	0.134 (−0.224 to 0.493)	0.463
Mixed	0.421 (−0.026 to 0.868)	0.065	0.522 (0.072 to 0.971)	0.023
Other	1.614 (1.027 to 2.200)	<0.001	2.072 (1.468 to 2.675)	<0.001
Fee status				
Home	1		1	
European Union	−0.741 (−1.068 to −0.413)	<0.001	−0.374 (−0.712 to −0.035)	0.030
Overseas	−0.087 (−0.379 to 0.206)	0.562	−0.052 (−0.345 to 0.241)	0.730
Disability				
Yes	1.147 (0.797 to 1.497)	<0.001	1.155 (0.802 to 1.508)	<0.001
No	1		1	

CORE-OM, Clinical Outcomes in Routine Evaluation.

**Table 4:** Linear regression analysis on the association between potential explanatory factors and CORE-OM risk score (*n* = 9517)

	Unadjusted		Fully adjusted	
Fixed effects	β or mean difference (95% CI)	*P*	β or mean difference (95% CI)	*P*
Age	−0.081 (−0.095 to −0.067)	<0.001	−0.078 (−0.092 to −0.063)	<0.001
Gender				
Male	1		1	
Female	0.059 (−0.101 to 0.219)	0.471	0.020 (−0.138 to 0.179)	0.802
Other	1.460 (0.763 to 2.158)	<0.001	0.884 (0.187 to 1.581)	0.013
Sexual orientation				
Heterosexual	1		1	
Bisexual	1.210 (1.007 to 1.413)	<0.001	1.121 (0.917 to 1.325)	<0.001
Gay/lesbian	0.521 (0.214 to 0.829)	0.001	0.538 (0.226 to 0.848)	0.001
Not sure/queer	0.809 (0.584 to 1.035)	<0.001	0.726 (0.501 to 0.952)	<0.001
Ethnicity				
Black	0.168 (−0.126 to 0.461)	0.263	0.286 (−0.006 to 0.578)	0.054
South Asian	0.393 (0.185 to 0.602)	<0.001	0.481 (0.268 to 0.694)	<0.001
Chinese	0.987 (0.731 to 1.243)	<0.001	1.21 (0.920 to 1.510)	<0.001
Other Asian	0.651 (0.366 to 0.936)	<0.001	0.836 (0.542 to 1.130)	<0.001
White British	1		1	
Other White	−0.280 (−0.464 to −0.095)	0.003	−0.130 (−0.332 to 0.071)	0.206
Mixed	0.223 (−0.030 to 0.475)	0.084	0.235 (−0.018 to 0.488)	0.068
Other	0.405 (0.074 to 0.736)	0.017	0.660 (0.320 to 0.999)	<0.001
Fee status				
Home	1		1	
European Union	−0.443 (−0.627 to −0.258)	<0.001	−0.308 (−0.498 to −0.118)	0.001
Overseas	0.088 (−0.077 to 0.253)	0.296	0.145 (−0.019 to 0.310)	0.083
Disability				
Yes	0.482 (0.284 to 0.680)	<0.001	0.545 (0.347 to 0.743)	<0.001
No	1		1	

CORE-OM, Clinical Outcomes in Routine Evaluation.

**Table 5:** Unadjusted and adjusted linear regression analysis on the association between potential explanatory factors and CIAO total score (*n* = 9517)

	Unadjusted		Fully adjusted	
Fixed effects	β/mean difference (95% CI)	*P*	β/mean difference (95% CI)	*P*
Age	0.013 (0.001 to 0.025)	0.033	0.011 (−0.001 to 0.023)	0.074
Gender				
Male	1		1	
Female	−0.098 (−0.235 to 0.039)	0.016	−0.098 (−0.235 to 0.038)	0.157
Other	0.583 (−0.014 to 10.180)	0.055	0.415 (−0.185 to 10.016)	0.175
Sexual orientation				
Heterosexual	1		1	
Bisexual	0.109 (−0.066 to 0.285)	0.221	0.126 (−0.050 to 0.302)	0.162
Gay/lesbian	−0.095 (−0.361 to 0.170)	0.481	−0.117 (−0.385 to 0.152)	0.395
Not sure/queer	0.052 (−0.143 to 0.247)	0.601	0.065 (−0.129 to 0.260)	0.510
Ethnicity				
Black	0.682 (0.431 to 0.933)	0.000	0.766 (0.515 to 10.017)	0.000
South Asian	0.424 (0.245 to 0.602)	0.000	0.645 (0.462 to 0.829)	0.000
Chinese	0.080 (−0.139 to 0.300)	0.473	0.637 (0.383 to 0.890)	0.000
Other Asian	0.563 (0.319 to 0.808)	0.000	0.880 (0.627 to 10.133)	0.000
White British	1		1	
Other White	−0.104 (−0.262 to 0.054)	0.195	0.222 (0.048 to 0.396)	0.012
Mixed	0.091 (−0.126 to 0.307)	0.411	0.231 (0.013 to 0.449)	0.037
Other	0.766 (0.482 to 10.050)	0.000	10.088 (0.796 to 10.380)	0.000
Fee status				
Home	1		1	
European Union	−0.403 (−0.561 to −0.245)	0.000	−0.252 (−0.415 to −0.088)	0.003
Overseas	−0.375 (−0.516 to −0.234)	0.000	−0.342 (−0.483 to −0.200)	0.000
Disability				
Yes	0.789 (0.621 to 0.958)	0.000	0.729 (0.559 to 0.900)	0.000
No	1		1	

CIAO, Counselling Impact on Academic Outcomes.

Younger students had higher psychological distress (*β* = −0.067, 95% CI −0.093 to −0.042) and clinical risk (*β* = −0.078, 95% CI −0.092 to −0.063) compared with their older counterparts.

Students who identified as female had higher psychological distress (mean difference 0.746, 95% CI 0.463–1.028) compared with male students. Those who identified as non-binary/genderqueer also had higher psychological distress (mean difference 1.488, 95% CI 0.246–2.731) and clinical risk (mean difference 0.884, 95% CI 0.187–1.581) compared with male students.

Gay/lesbian students had higher clinical risk (mean difference 0.538, 95% CI 0.226–0.848) than heterosexual students. Bisexual and not sure/queer students had higher psychological distress (mean difference 0.830, 95% CI 0.466–1.194 and mean difference 0.789, 95% CI 0.386–1.192 respectively) and clinical risk (mean difference 1.121, 95% CI 0.917–1.325 and mean difference 0.726, 95% CI 0.501–0.952 respectively) compared with heterosexual counterparts.

Compared to their White counterparts, Black (mean difference 1.350, 95% CI 0.831–1.869), South Asian (mean difference 1.715, 95% CI 1.336–2.095), Chinese (mean difference 1.740, 95% CI 1.215–2.264), Other Asian (mean difference 2.062, 95% CI 1.540–2.585), Mixed (mean difference 0.522, 95% CI 0.072–0.971) and Other ethnic group (mean difference 2.072, 95% CI 1.468–2.675) students had higher psychological distress, South Asian (mean difference 0.481, 95% CI 0.268–0.694), Chinese (mean difference 1.21, 95% CI 0.920–1.510), Other Asian (mean difference 0.836, 95% CI 0.542–1.130) and Other ethnic group (mean difference 0.660, 95% CI 0.320–0.999) students had higher clinical risk and Black (mean difference 0.766, 95% CI 0.515–1.017), South Asian (mean difference 0.645, 95% CI 0.462–0.829), Chinese (mean difference 0.637, 95% CI 0.383–0.890), Other Asian (mean difference 0.880, 95% CI 0.627–1.133), Other White (mean difference 222, 95% CI 0.048–0.396), Mixed (mean difference 0.231, 95% CI 0.013–0.449) and Other ethnic group (mean difference 1.088, 95% CI 0.796–1.380) students had higher impact of problems on academic outcomes.

Students with EU fee status had lower psychological distress (mean difference −0.374, 95% CI −0.712 to −0.035), clinical risk (mean difference −0.308, 95% CI −0.498 to −0.118) and impact of problems on academic outcomes (mean difference −0.252, 95% CI −0.415 to −0.088) compared with students with home fee status. Overseas students also had lower impact of problems on academic outcomes (mean difference −0.342, 95% CI −0.483 to −0.200) than those with home fee status.

Those registered with a disability had higher psychological distress (mean difference 1.155, 95% CI 0.802–1.508), clinical risk (mean difference 0.545, 95% CI 0.347–0.743) and impact of problems on academic outcomes (mean difference 0.729, 95% CI 0.559–0.900) compared with those without.

### Sensitivity analyses

When the pandemic (pre- versus peri-pandemic) time points were broken down into academic years (2018–2019, 2019–2020, 2020–2021 and 2021–2022), *post hoc* Tukey tests found no significant differences between outcomes in individual academic years during the pandemic (Supplementary Table 2).

Linear regression models conducted on the pre- and peri-pandemic samples separately mainly found no differences (Supplementary Tables 3–5). However, students who identified as non-binary/genderqueer did not have higher psychological distress than male counterparts in the pre-pandemic sample or have higher clinical risk than male students in peri-pandemic. Other ethnic group students did not have higher psychological distress than White students in the pre-pandemic sample. Lastly, Other White and Mixed ethnic group students did not have higher impact of problems on academic outcomes than White students in the pre- or peri-pandemic samples. Multiple imputation analyses used a sample of 9616. Multiply imputed adjusted analyses were not different from complete cases across all outcomes (Supplementary Tables 6–8).

## Discussion

### Main findings

This study aimed to investigate (a) how psychological distress, clinical risk and impact of problems on academic outcomes differed before and during the COVID-19 pandemic; and (b) potential explanatory factors for psychological distress, clinical risk and impact of problems on academic outcomes in university students at service entry using routine data from a CMHS service.

Comparison of pre- and peri-pandemic samples suggests that there were no significant differences between psychological distress and impact of problems on academic outcomes in students presenting to the service with mental health problems. These findings are similar to other studies which found little change in general mental health outcomes between pre- and peri-pandemic time points in university students.^20^ Our findings suggest clinical risk was significantly higher pre-pandemic compared with peri-pandemic, which contrasts with previous findings on increased prevalences of suicidal ideation and self-harm in student and adult samples.^2,21–23^

Younger age was identified as significantly associated with higher psychological distress and clinical risk. Younger students may face several additional but universal challenges, which may explain why they present to the service with worse mental health outcomes; the challenges include managing the transition to university from school, moving away from home, losing familiar support networks, and still developing self-efficacy and socioemotional regulation skills. Although some studies suggest that psychological distress declines as age increases,^25^ it is unclear whether the reduction of psychological distress is related to temporal or generational changes, whereby younger students may have higher psychological distress compared with older students by default, because of when they were born.

Female gender was associated with higher psychological distress. This complements existing research that female students have worse mental health outcomes compared with male counterparts, which may be due to caring responsibilities, disproportionate loss of employment and other factors.^26–28^ Non-binary/genderqueer students were also more likely to report higher psychological distress and clinical risk than male students, which could be due to increased likelihood of childhood abuse or assault in relation to being gender non-conforming.^29^ Although our study adds to the gender literature, as many studies treat gender as a binary variable, the number of non-binary/genderqueer students was very small so our finding should be carefully interpreted.

We found that being gay/lesbian was significantly associated with higher clinical risk and being bisexual and not sure/queer was significantly associated with higher psychological distress and clinical risk. This adds to the literature that LGBT people report more mental health problems than heterosexual adults, and bisexual and other queer people may have the worst outcomes.^30,31^ This may be due to stigma and discrimination from heterosexual communities and within the queer communities. Importantly, our findings add to the sparse evidence on sexual orientation and mental health outcomes in UK university students, as most studies use general adult samples.

Asian, Black and Mixed ethnicity were significantly associated with increased psychological distress, clinical risk and impact of problems on academic outcomes. This has already been reflected in previous studies, which have suggested that experiences of structural racism and disproportionate material/academic hardship may be a reason for worse mental health outcomes in these groups.^32,33^ Those who experienced perceived racism were also more likely to have trouble with executive functioning,^32^ which may affect academic outcomes. Our findings add to the existing literature that students from minority ethnic groups have worse mental health outcomes and provide more detailed breakdowns of mental health outcomes between ethnic groups than most studies are unable to do.^34,35^

Being registered with a disability was significantly associated with increased psychological distress, clinical risk and impact of problems on academic outcomes. To our knowledge, no studies have shown this in a large UK university student sample, so our findings provide evidence for the disproportionate mental health needs of students with a disability. Potential reasons for the worse mental health and academic outcomes than those without disability may be poorer social connectedness, lack of social support, reduced self-efficacy and feeling not supported in their learning environment.^36,37^

Home fee status was significantly associated with increased psychological distress and impact of problems on academic outcomes. This replicates other studies which found that home/domestic students reported worse mental health outcomes than overseas/international students.^38,39^ However, the only UK study used a small sample and did not adjust for potential confounding.^39^ Although reasons for this disparity in outcomes are still unclear, possible explanations span from cultural stigma and reporting artefact to socioeconomic status and disproportionate resource provision for acculturation of international students.

Most of the explanatory factors were significantly associated with increased psychological distress, clinical risk and impact of problems on academic outcomes in both the pre- and peri-pandemic groups, suggesting that explanatory factors did not differ much between the pre- and peri-pandemic time points. This elucidates that these key explanatory factors are stably associated with poor mental health and academic outcomes in university students across time. This adds to our current understanding of risk factors for poor mental health in university students generally and in the context of the pandemic.^40^ This may suggest that pre-existing risk factors continue to play a role in shaping university student outcomes during the pandemic. However, the causal relationship between the pandemic and university student outcomes still needs further research.

### Strengths and limitations

This study uses routine data of 9517 students across pre- and peri-pandemic time points who presented to a CMHS service. We include a wide range of important sociodemographic characteristics that many studies do not assess, including gender and sexual minorities, finer categories of ethnicity, fee status and disability registration. We were able to look across characteristics and adjust for them in analyses to provide stronger evidence on their associations with university student mental health and academic outcomes. Our data are largely complete and the proportion of missing measurements of sociodemographic and outcome data are mostly similar across characteristics and time points. This adds to the current literature and suggests potential risk factors that could be used to identify students who may need support. Our study emphasises the need to support groups of university students who may be at higher risk of poorer mental health and academic outcomes. In addition to psychological distress and clinical risks, we also assess impact on academic outcomes to understand the potential impact of mental health problems in university students and their experiences.

However, there are several limitations to our study. First, we could not assess some potential explanatory factors, such as socioeconomic and income factors, trans identity, pre-existing mental illness, educational attainment and course characteristics (e.g. mode and level of study), as data were not available. Second, we could not examine changes in mental health and academic outcomes longitudinally during the pandemic owing to the repeated cross-sectional design of our study. Third, our cohort captures only students who presented to the CMHS service. It therefore does not include students who did not access mental health support for whatever reason and it may not include students experiencing milder mental health problems. This potential sampling bias should be considered when interpreting our findings as these findings do not reflect population-level changes in student mental health before and during the pandemic.

### Implications

Our findings prompt future research to parse causal pathways in poor university student mental health. The mechanisms of changes in psychological distress, clinical risk and impact of problems on academic outcomes is still unclear. Future research should explore the intersection of multiple social identities and how they interact in student mental health and academic outcomes. With emerging advanced statistical analyses using intersectional frameworks, intersectionality should be considered when investigating causal pathways in university mental health to inform enhanced and individualised care. Data post-July 2022 and end-of-treatment outcomes should also be analysed to examine potential longitudinal impacts of the pandemic on student mental health and the impact of the support offered by universities. In-depth qualitative studies could also be used to explore potential mechanisms of change in university student mental health.

Understanding what individual characteristics may be associated with worse psychological distress, clinical risk and impact of problems on academic outcomes may inform targeted outreach and support to those who may be more vulnerable. Methods such as digital and social media outreach, whole university campaigns or large-scale events could promote the use of CMHS services to university students and encourage uptake by specific populations.

Universities should ensure that strategies are implemented to reduce inequities that may be contributing to poorer mental health and academic outcomes. Emerging ideas of achieving this include integrating inclusive and culturally responsive pedagogy into curricula, establishing safe spaces for minority groups for community and support, clear reporting systems and processes for students who experience discrimination, and training and awareness campaigns.

We need to better understand how universities and student CMHS services can most efficiently and effectively support the mental health needs of university students. As the long-term consequences of the pandemic on university student mental health and academic outcomes are not fully established, it is crucial to ensure that systems are equipped to offer support to university students who need it.

## Supporting information

Ching et al. supplementary material 1 Ching et al. supplementary material

Ching et al. supplementary material 2 Ching et al. supplementary material

Ching et al. supplementary material 3 Ching et al. supplementary material

Ching et al. supplementary material 4 Ching et al. supplementary material

Ching et al. supplementary material 5 Ching et al. supplementary material

Ching et al. supplementary material 6 Ching et al. supplementary material

Ching et al. supplementary material 7 Ching et al. supplementary material

Ching et al. supplementary material 8 Ching et al. supplementary material

## Data Availability

The data are not publicly available but may be available on reasonable request from the corresponding author, A.A.

## Supplementary material

The supplementary material is available online at https://doi.org/10.1192/bjo.2024.868.

## References

[1] Auerbach RP , Mortier P , Bruffaerts R , Alonso J , Benjet C , Cuijpers P , et al. WHO World Mental Health Surveys International College Student Project: prevalence and distribution of mental disorders. J Abnormal Psychol 2018; 127: 623–38. 10.1037/abn0000362 PMC6193834 30211576

[2] Tang NK , McEnery KA , Chandler L , Toro C , Walasek L , Friend H , et al. Pandemic and student mental health: mental health symptoms among university students and young adults after the first cycle of lockdown in the UK. BJPsych Open 2022; 8: e138. 35880308 10.1192/bjo.2022.523 PMC9345288

[3] Evans S , Alkan E , Bhangoo JK , Tenenbaum H , Ng-Knight T. Effects of the COVID-19 lockdown on mental health, wellbeing, sleep, and alcohol use in a UK student sample. Psychiatry Res 2021; 298: 113819. 33640864 10.1016/j.psychres.2021.113819 PMC9754711

[4] Savage MJ , James R , Magistro D , Donaldson J , Healy LC , Nevill M , et al. Mental health and movement behaviour during the COVID-19 pandemic in UK university students: prospective cohort study. Ment Health Phys Activity 2020; 19: 100357.

[5] Wathelet M , Duhem S , Vaiva G , Baubet T , Habran E , Veerapa E , et al. Factors associated with mental health disorders among university students in France confined during the COVID-19 pandemic. JAMA Netw Open 2020; 3: e2025591. 33095252 10.1001/jamanetworkopen.2020.25591 PMC7584927

[6] Gewalt SC , Berger S , Krisam R , Breuer M. Effects of the COVID-19 pandemic on university students’ physical health, mental health and learning, a cross-sectional study including 917 students from eight universities in Germany. PLoS One 2022; 17: e0273928. 36044521 10.1371/journal.pone.0273928 PMC9432688

[7] Matos Fialho PM , Spatafora F , Kühne L , Busse H , Helmer SM , Zeeb H , et al. Perceptions of study conditions and depressive symptoms during the COVID-19 pandemic among university students in Germany: Results of the international COVID-19 student well-being study. Front Publ Health 2021; 9: 674665. 10.3389/fpubh.2021.674665 PMC8222519 34178930

[8] Sampson K , Priestley M , Dodd AL , Broglia E , Wykes T , Robotham D , et al. Key questions: research priorities for student mental health. BJPsych Open 2022; 8: e90. 35535504 10.1192/bjo.2022.61 PMC9169497

[9] Prowse R , Sherratt F , Abizaid A , Gabrys RL , Hellemans KG , Patterson ZR , et al. Coping with the COVID-19 pandemic: examining gender differences in stress and mental health among university students. Front Psychiatry 2021; 12: 650759. 33897499 10.3389/fpsyt.2021.650759 PMC8058407

[10] Lin SY , Schleider JL , Nelson BD , Richmond LL , Eaton NR. Gender and racial/ethnic disparities in undergraduate and graduate students’ mental health and treatment use amid the COVID-19 pandemic. Adm Policy Ment Health 2023; 50: 552–62. 36802042 10.1007/s10488-023-01256-z PMC9937864

[11] Aguilar O , Lipson SK. A public health approach to understanding the mental health needs of college students with disabilities: results from a national survey. J Postsecond Educ Disab 2021; 34: 273–85.

[12] Grimes S , Southgate E , Scevak J , Buchanan R. Learning impacts reported by students living with learning challenges/disability. Stud High Educ 2021; 46: 1146–58.

[13] Lett K , Tamaian A , Klest B. Impact of ableist microaggressions on university students with self-identified disabilities. Disab Soc 2020; 35: 1441–56.

[14] Gonzales G , de Mola EL , Gavulic KA , McKay T , Purcell C. Mental health needs among lesbian, gay, bisexual, and transgender college students during the COVID-19 pandemic. J Adolesc Health 2020; 67: 645–8. 32933837 10.1016/j.jadohealth.2020.08.006 PMC10251764

[15] Salerno JP , Shrader CH , Algarin AB , Lee JY , Fish JN. Changes in alcohol use since the onset of COVID-19 are associated with psychological distress among sexual and gender minority university students in the US. Drug Alcohol Depend 2021; 221: 108594. 33689965 10.1016/j.drugalcdep.2021.108594 PMC8104058

[16] Evans C , Connell J , Barkham M , Margison F , McGrath G , Mellor-Clark J , et al. Towards a standardised brief outcome measure: Psychometric properties and utility of the CORE–OM. Br J Psychiatry 2002; 180: 51–60. 11772852 10.1192/bjp.180.1.51

[17] McInnes, B. Made to Measure: CORE. Therapy Meets Numbers, 2018 (https://therapymeetsnumbers.com/made-to-measure-core/).

[18] Core IMS. CORE System User Manual. Core IMS, 2016.

[19] Wallace P. The impact of counselling on academic outcomes: the student perspective. AUCC J 2012; 7: 6–11.

[20] Sun Y , Wu Y , Fan S , Dal Santo T , Li L , Jiang X , et al. Comparison of mental health symptoms before and during the Covid-19 pandemic: evidence from a systematic review and meta-analysis of 134 cohorts. BMJ 2023; 380: 1–10. 36889797 10.1136/bmj-2022-074224 PMC9992728

[21] Farooq S , Tunmore J , Ali MW , Ayub M. Suicide, self-harm and suicidal ideation during COVID-19: a systematic review. Psychiatry Res 2021; 306: 114228. 34670162 10.1016/j.psychres.2021.114228 PMC8495045

[22] Ma Z , Wang D , Zhao J , Zhu Y , Zhang Y , Chen Z , et al. Longitudinal associations between multiple mental health problems and suicidal ideation among university students during the COVID-19 pandemic. J Affect Disord 2022; 311: 425–31. 35597475 10.1016/j.jad.2022.05.093 PMC9116974

[23] Sivertsen B , Knapstad M , Petrie K , O’Connor R , Lonning KJ , Hysing M. Changes in mental health problems and suicidal behaviour in students and their associations with COVID-19 related restrictions in Norway: a national repeated cross-sectional analysis. BMJ Open 2022; 12: e057492. 10.1136/bmjopen-2021-057492 PMC8829843 35140162

[24] Carey EG , Ridler I , Ford TJ , Stringaris A. Editorial perspective: when is a ‘small effect’ actually large and impactful? J Child Psychol Psychiatry 2023; 64: 1643–7. 37226639 10.1111/jcpp.13817

[25] Charles ST , Rush J , Piazza JR , Cerino ES , Mogle J , Almeida DM. Growing old and being old: emotional well-being across adulthood. J Pers Soc Psychol 2023; 125: 455–69. 36848104 10.1037/pspp0000453 PMC10330366

[26] Breslau J , Roth EA , Baird MD , Carman KG , Collins RL. A longitudinal study of predictors of serious psychological distress during COVID-19 pandemic. Psycholog Med 2023; 53: 2418–26. 10.1017/S0033291721004293 PMC8523967 34629132

[27] Collins C , Landivar LC , Ruppanner L , Scarborough WJ. COVID-19 and the gender gap in work hours. Gender Work Org 2021; 28: 101–12. 10.1111/gwao.12506 PMC7361447 32837019

[28] Victor SE , Muehlenkamp JJ , Hayes NA , Lengel GJ , Styer DM , Washburn JJ. Characterizing gender differences in nonsuicidal self-injury: evidence from a large clinical sample of adolescents and adults. Compr Psychiatry 2018; 82: 53–60. 29407359 10.1016/j.comppsych.2018.01.009 PMC5845831

[29] Rimes KA , Goodship N , Ussher G , Baker D , West E. Non-binary and binary transgender youth: comparison of mental health, self-harm, suicidality, substance use and victimization experiences. In Non-Binary and Genderqueer Genders (eds M Joz, T Nieder, W Bouman): 112–22. Routledge, 2020. 10.1080/15532739.2017.1370627 PMC6831005 32999609

[30] Buspavanich P , Lech S , Lermer E , Fischer M , Berger M , Vilsmaier T , et al. Well-being during COVID-19 pandemic: a comparison of individuals with minoritized sexual and gender identities and cis-heterosexual individuals. PLoS One 2021; 16: e0252356. 34101746 10.1371/journal.pone.0252356 PMC8186787

[31] Dunlop BJ , Hartley S , Oladokun O , Taylor PJ. Bisexuality and non-suicidal self-injury (NSSI): a narrative synthesis of associated variables and a meta-analysis of risk. J Affect Disord 2020; 276: 1159–72. 32823255 10.1016/j.jad.2020.07.103

[32] Mpofu JJ , Cooper AC , Ashley C , Geda S , Harding RL , Johns MM , et al. Perceived racism and demographic, mental health, and behavioral characteristics among high school students during the COVID-19 pandemic – Adolescent Behaviors and Experiences Survey, United States, January–June 2021. MMWR Suppl 2022; 71: 22. 35358163 10.15585/mmwr.su7103a4 PMC8979604

[33] Jabbari J , Ferris D , Frank T , Malik S , Bessaha M. Intersecting race and gender across hardships and mental health during COVID-19: a moderated-mediation model of graduate students at two universities. Race Soc Probl 2023; 15: 328–46. 10.1007/s12552-022-09379-y PMC9595585 36313213

[34] Proto E , Quintana-Domeque C. COVID-19 and mental health deterioration by ethnicity and gender in the UK. PLoS One 2021; 16: e0244419. 33406085 10.1371/journal.pone.0244419 PMC7787387

[35] Chen T , Lucock M. The mental health of university students during the COVID-19 pandemic: an online survey in the UK. PLoS One 2022; 17: e0262562. 35020758 10.1371/journal.pone.0262562 PMC8754313

[36] Dryer R , Henning MA , Tyson GA , Shaw R. Academic achievement performance of university students with disability: exploring the influence of non-academic factors. Int J Disabil Develop Educ 2016; 63: 419–30.

[37] McManus D , Dryer R , Henning M. Barriers to learning online experienced by students with a mental health disability. Dist Educ 2017; 38: 336–52.

[38] King N , Rivera D , Cunningham S , Pickett W , Harkness K , McNevin SH , et al. Mental health and academic outcomes over the first year at university in international compared to domestic Canadian students. J Am College Health 2023; 71: 2663–72. 10.1080/07448481.2021.1982950 34606410

[39] Jones CP , Lodder A , Papadopoulos C. Do predictors of mental health differ between home and international students studying in the UK? J Appl Res High Educ 2019; 11: 224–34.

[40] Xu Y , Su S , Jiang Z , Guo S , Lu Q , Liu L , et al. Prevalence and risk factors of mental health symptoms and suicidal behavior among university students in Wuhan, China during the COVID-19 pandemic. Front Psychiatry 2021; 12: 695017. 34326787 10.3389/fpsyt.2021.695017 PMC8313758

