# Supplementary material for: Poor mental health and its impact on academic outcomes in university students before and during the COVID-19 pandemic: analysis of routine service data
Source: BJPsych Open. 2025 Mar 11;11(2):e46. doi: 10.1192/bjo.2024.868 (PMC12001929; doi:10.1192/bjo.2024.868)
Supplement: Ching et al. supplementary material 1 — Ching et al. supplementary material [file S2056472424008688sup001.docx]

Supplementary Table 1. Sample characteristics of students with complete or missing sociodemographic and outcome data.

|  | **Whole cohort**  (n = 10,055) | | **Pre-pandemic**  (n = 4,666) | | **Peri-pandemic**  (n = 5,389) | |
| --- | --- | --- | --- | --- | --- | --- |
|  | Complete | Missing | Complete | Missing | Complete | Missing |
| n (%) | (n = 9,517) | (n = 538) | (n = 4,522) | (n = 144) | (n = 4,995) | (n = 394) |
| Age |  |  |  |  |  |  |
| 16-24 | 7,290 (94.79) | 401 (5.21) | 3,520 (96.94) | 111 (3.06) | 3,769 (92.86) | 290 (7.14) |
| 25-34 | 1,962 (94.60) | 112 (5.40) | 873 (97.22) | 25 (2.78) | 1,089 (92.60) | 87 (7.40) |
| 35≤ | 265 (91.38) | 25 (8.62) | 128 (94.12) | 8 (5.88) | 137 (88.96) | 17 (11.04) |
| Gender |  |  |  |  |  |  |
| Male | 2,051 (94.17) | 127 (5.83) | 990 (96.87) | 32 (3.13) | 1,061 (91.78) | 95 (8.22) |
| Female | 7,378 (94.77) | 407 (5.23) | 3,509 (96.96) | 110 (3.04) | 3,869 (92.87) | 297 (7.13) |
| Other | 88 (95.65) | 4 (4.35) | 23 (92.00) | 2 (8.00) | 65 (97.01) | 2 (2.99) |
| Sexual orientation |  |  |  |  |  |  |
| Heterosexual | 7,029 (94.37) | 419 (5.63) | 3,442 (96.82) | 113 (3.18) | 3,587 (92.14) | 306 (7.86) |
| Bisexual | 1,140 (95.88) | 49 (4.12) | 482 (97.57) | 12 (2.43) | 658 (94.68) | 37 (5.32) |
| Gay/lesbian | 454 (95.58) | 21 (4.42) | 219 (96.90) | 7 (3.10) | 235 (94.38) | 14 (5.62) |
| Not sure/queer | 894 (94.80) | 49 (5.20) | 379 (96.93) | 12 (3.07) | 515 (93.30) | 37 (6.70) |
| Ethnicity |  |  |  |  |  |  |
| Black | 567 (94.82) | 31 (5.18) | 245 (98.39) | 4 (1.61) | 322 (92.26) | 27 (7.74) |
| South Asian | 1,397 (94.97) | 74 (5.03) | 623 (97.04) | 19 (2.96) | 774 (93.37) | 55 (6.63) |
| Chinese | 792 (91.88) | 70 (8.12) | 338 (97.69) | 8 (2.31) | 454 (87.98) | 62 (12.02) |
| Other Asian | 608 (95.45) | 29 (4.55) | 276 (97.87) | 6 (2.13) | 332 (93.52) | 23 (6.48) |
| White British | 2,829 (95.06) | 147 (4.94) | 1,408 (97.04) | 43 (2.96) | 1,421 (93.18) | 104 (6.82) |
| Other White | 2,076 (94.58) | 119 (5.42) | 1,044 (96.13) | 42 (3.87) | 1,032 (93.06) | 77 (6.94) |
| Mixed | 822 (95.80) | 36 (4.20) | 390 (96.77) | 13 (3.23) | 432 (94.95) | 23 (5.05) |
| Other | 426 (93.01) | 32 (6.99) | 198 (95.65) | 9 (4.35) | 228 (90.84) | 23 (9.16) |
| Fee status |  |  |  |  |  |  |
| Home | 5,994 (95.10) | 309 (4.90) | 2,776 (97.20) | 80 (2.80) | 3,218 (93.36) | 229 (6.64) |
| EU | 1,507 (94.54) | 87 (5.46) | 785 (96.08) | 32 (3.92) | 722 (92.92) | 55 (7.08) |
| Overseas | 2,016 (93.42) | 142 (6.58) | 961 (96.78) | 32 (3.22) | 1,055 (90.56) | 110 (9.44) |
| Disability |  |  |  |  |  |  |
| Yes | 1,202 (94.05) | 76 (5.95) | 620 (96.12) | 25 (3.88) | 582 (91.94) | 51 (8.06) |
| No | 8,315 (94.74) | 462 (5.26) | 3,902 (97.04) | 119 (2.96) | 4,413 (92.79) | 343 (7.21) |

*Complete is when respondents have all sociodemographic and outcome variables available.

^Missingness is when respondents have at least one sociodemographic or outcome variable missing.
