# Supplementary material for: Poor mental health and its impact on academic outcomes in university students before and during the COVID-19 pandemic: analysis of routine service data
Source: BJPsych Open. 2025 Mar 11;11(2):e46. doi: 10.1192/bjo.2024.868 (PMC12001929; doi:10.1192/bjo.2024.868)
Supplement: Ching et al. supplementary material 2 — Ching et al. supplementary material [file S2056472424008688sup002.docx]

Supplementary Table 2. Sensitivity analysis: one-way ANOVA and post-hoc Tukey tests comparing CORE-OM total, CORE-OM risk, and CIAO total scores across academic years (n = 9,517).

|  |  | 2018-2019 vs 2019-2020 | 2018-2019 vs 2020-2021 | 2018-2019 vs 2021-2022 | 2019-2020 vs 2020-2021 | 2019-2020 vs 2021-2022 | 2020-2021 vs 2021-2022 |
| --- | --- | --- | --- | --- | --- | --- | --- |
| CORE-OM total | F(3, 9513) = 4.31, p = .005 | -.446 ± .16,  p = .029,  95% CI [-.860 to -.031] | -.391 ± .16,  p = .080,  95% CI [-.813 to .030] | -.395 ± .16,  p = .067,  95% CI [-.808 to 0.018] | .055 ± .16,  p = .987,  95% CI [-.369 to .478] | .051 ± .16,  p = .989,  95% CI [-.364 to .466] | -.004 ± .16,  p = 1.000,  95% CI [-.426 to .418] |
| CORE-OM risk | F(3, 9513) = 4.13, p = .006 | -.238 ± .09,  p = .046,  95% CI [-.472 to -.003] | -.300 ± .09,  p = .007,  95% CI [-.539 to -.061] | -.234 ± .09,  p = .049,  95% CI [-.468 to -.000] | -.062 ± .09,  p = .911,  95% CI [-.301 to .178] | .004 ± .09,  p = 1.000,  95% CI [-.231 to .238] | .066 ± .09,  p = .895,  95% CI -.173 to .304] |
| CIAO total | F(3, 9513) = 7.21, p = .000 | -.340 ± .08,  p = .000,  95% CI [-.540 to -.140] | -.278 ± .08,  p = .002,  95% CI [-.481 to -.075] | -.243 ± .08,  p = .009,  95% CI [-.442 to -.044] | .062 ± .08,  p = .863,  95% CI [-.142 to .266] | .097 ± .08,  p = .600,  95% CI [-.103 to .297] | .035 ± .08,  p = .097,  95% CI [-.168 to .237] |
