# Supplementary material for: Poor mental health and its impact on academic outcomes in university students before and during the COVID-19 pandemic: analysis of routine service data
Source: BJPsych Open. 2025 Mar 11;11(2):e46. doi: 10.1192/bjo.2024.868 (PMC12001929; doi:10.1192/bjo.2024.868)
Supplement: Ching et al. supplementary material 3 — Ching et al. supplementary material [file S2056472424008688sup003.docx]

Supplementary Table 3. Unadjusted and adjusted linear regression analysis on the association between potential explanatory factors and CORE-OM total score in the pre- and peri-pandemic samples (n = 9,517).

|  | **Unadjusted** | | | | **Fully adjusted** | | | |
| --- | --- | --- | --- | --- | --- | --- | --- | --- |
|  | Pre-pandemic | | Peri-pandemic | | Pre-pandemic | | Peri-pandemic | |
| **Fixed effects** | β/mean difference (95% CI) | p | β/mean difference (95% CI) | p | β/mean difference (95% CI) | p | β/mean difference (95% CI) | p |
| Age | -.046 (-.083 to -.010) | .012 | -.096 (-.131 to -.061) | .000 | -.045 (-.081 to -.009) | .016 | -.087 (-.122 to -.051) | .000 |
| Gender |  |  |  |  |  |  |  |  |
| Male | 1 |  | 1 |  | 1 |  | 1 |  |
| Female | .930 (.520 to 1.339) | .000 | .691 (.299 to 1.084) | .001 | .878 (.469 to 1.288) | .000 | .622 (.232 to 1.012) | .002 |
| Other | 1.43 (-.976 to 3.827) | .245 | 2.344 (.897 to 3.791) | .002 | .949 (-1.462 to 3.359) | .440 | 1.620 (.164 to 3.076) | .029 |
| Sexual orientation |  |  |  |  |  |  |  |  |
| Heterosexual | 1 |  | 1 |  | 1 |  | 1 |  |
| Bisexual | .874 (.320 to 1.428) | .002 | 1.022 (.542 to 1.501) | .000 | .795 (.240 to 1.350) | .005 | .874 (.390 to 1.359) | .000 |
| Gay/lesbian | -.709 (-1.503 to .084) | .080 | -.058 (-.820 to .704) | .881 | -.288 (-1.095 to .519) | .485 | .071 (-.696 to .838) | .857 |
| Not sure/queer | .810 (.193 to 1.426) | .010 | 1.013 (.480 to 1.546) | .000 | .723 (.108 to 1.338) | .021 | .862 (.327 to 1.396) | .002 |
| Ethnicity |  |  |  |  |  |  |  |  |
| Black | 1.200 (.416 to 1.983) | .003 | 1.368 (.673 to 2.063) | .000 | 1.210 (.423 to 1.997) | .003 | 1.461 (.769 to 2.152) | .000 |
| South Asian | 1.420 (.876 to 1.965) | .000 | 1.541 (1.038 to 2.044) | .000 | 1.523 (.965 to 2.080) | .000 | 1.929 (1.409 to 2.449) | .000 |
| Chinese | 1.618 (.932 to 2.304) | .000 | .827 (.220 to 1.434) | .008 | 1.948 (1.155 to 2.742) | .000 | 1.663 (.962 to 2.365) | .000 |
| Other Asian | 1.849 (1.104 to 2.594) | .000 | 1.631 (.944 to 2.317) | .000 | 2.019 (1.244 to 2.794) | .000 | 2.129 (1.422 to 2.837) | .000 |
| White British | 1 |  | 1 |  | 1 |  | 1 |  |
| Other White | -.248 (-.710 to .214) | .293 | -.203 (-.663 to .258) | .388 | -.058 (-.578 to .462) | .828 | .282 (-.215 to .779) | .265 |
| Mixed | .546 (-.101 to 1.194) | .098 | .318 (-.300 to .937) | .313 | .555 (-.099 to 1.208) | .096 | .496 (-.124 to 1.116) | .117 |
| Other | 1.344 (.485 to 2.203) | .002 | 1.863 (1.060 to 2.666) | .000 | 1.545 (.654 to 2.435) | .001 | 2.54 (1.722 to 3.366) | .000 |
| Fee status |  |  |  |  |  |  |  |  |
| Home | 1 |  | 1 |  | 1 |  | 1 |  |
| EU | -.692 (-1.153 to -.232) | .003 | -.799 (-1.265 to -.332) | .001 | -.692 (-1.153 to -.232) | .003 | -.440 (-.919 to .039) | .072 |
| Overseas | .300 (-.126 to .727) | .017 | -.440 (-.842 to -.038) | .032 | .300 (-.126 to .727) | .168 | -.388 (-.791 to .015) | .059 |
| Disability |  |  |  |  |  |  |  |  |
| Yes | 1.040 (.548 to 1.533) | .000 | 1.242 (.743 to 1.741) | .000 | 1.043 (0.547 to 1.539) | .000 | 1.250 (.747 to 1.754) | .000 |
| No | 1 |  | 1 |  | 1 |  | 1 |  |
