# Supplementary material for: Poor mental health and its impact on academic outcomes in university students before and during the COVID-19 pandemic: analysis of routine service data
Source: BJPsych Open. 2025 Mar 11;11(2):e46. doi: 10.1192/bjo.2024.868 (PMC12001929; doi:10.1192/bjo.2024.868)
Supplement: Ching et al. supplementary material 4 — Ching et al. supplementary material [file S2056472424008688sup004.docx]

Supplementary Table 4. Unadjusted and adjusted linear regression analysis on the association between potential explanatory factors and CORE-OM risk score in the pre- and peri-pandemic samples (n = 9,517).

|  | **Unadjusted** | | | | **Fully adjusted** | | | |
| --- | --- | --- | --- | --- | --- | --- | --- | --- |
|  | Pre-pandemic | | Peri-pandemic | | Pre-pandemic | | Peri-pandemic | |
| **Fixed effects** | β/mean difference (95% CI) | p | β/mean difference (95% CI) | p | β/mean difference (95% CI) | p | β/mean difference (95% CI) | p |
| Age | -.070 (-.090 to -.049) | .000 | -.092 (-.111 to -.072) | .000 | -.068 (-.088 to -.047) | .000 | -.086 (-.106 to -.067) | .000 |
| Gender |  |  |  |  |  |  |  |  |
| Male | 1 |  | 1 |  | 1 |  | 1 |  |
| Female | .054 (-.179 to .287) | .650 | .065 (-.154 to .285) | .560 | .028 (-.204 to .260) | .815 | .017 (-.200 to .234) | .876 |
| Other | 1.906 (.540 to 3.273) | .006 | 1.350 (.540 to 2.160) | .001 | 1.393 (.027 to 2.759) | .046 | .702 (-.108 to 1.511) | .089 |
| Sexual orientation |  |  |  |  |  |  |  |  |
| Heterosexual | 1 |  | 1 |  | 1 |  | 1 |  |
| Bisexual | 1.307 (.994 to 1.620) | .000 | 1.162 (.895 to 1.428) | .000 | 1.244 (.931 to 1.558) | .000 | 1.039 (.770 to 1.308) | .000 |
| Gay/lesbian | .509 (.061 to .957) | .026 | .536 (.113 to .959) | .013 | .610 (.154 to 1.066) | .009 | .470 (.044 to .895) | .031 |
| Not sure/queer | .656 (.308 to 1.004) | .000 | .944 (.648 to 1.241) | .000 | .599 (.252 to .947) | .001 | .831 (.534 to 1.127) | .000 |
| Ethnicity |  |  |  |  |  |  |  |  |
| Black | .193 (-.252 to .638) | .395 | .166 (-.225 to .556) | .406 | .343 (-.102 to .788) | .131 | .252 (-.134 to .637) | .201 |
| South Asian | .341 (.032 to .650) | .031 | .450 (.167 to .732) | .002 | .365 (.049 to .680) | .023 | .612 (.321 to .902) | .000 |
| Chinese | 1.171 (.782 to 1.561) | .000 | .869 (.528 to 1.209) | .000 | 1.277 (.828 to 1.726) | .000 | 1.213 (.822 to 1.604) | .000 |
| Other Asian | 1.101 (.678 to 1.524) | .000 | .289 (-.096 to .675) | .141 | 1.212 (.774 to 1.651) | .000 | .544 (.150 to .939) | .007 |
| White British | 1 |  | 1 |  | 1 |  | 1 |  |
| Other White | -.363 (-.626 to -.101) | .007 | -.196 (-.455 to .062) | .137 | -.281 (-.575 to .013) | .061 | .006 (-.271 to .284) | .964 |
| Mixed | .294 (-.074 to .662) | .117 | .165 (-.183 to .512) | .353 | .286 (-.084 to .656) | .130 | .201 (-.145 to .547) | .254 |
| Other | .292 (-.196 to .779) | .241 | .513 (.062 to .964) | .026 | .417 (-.087 to .920) | .105 | .882 (.423 to 1.340) | .000 |
| Fee status |  |  |  |  |  |  |  |  |
| Home | 1 |  | 1 |  | 1 |  | 1 |  |
| EU | -.502 (-.763 to -.240) | .000 | -.390 (-.651 to -.129) | .003 | -.351 (-.622 to -.079) | .011 | -.267 (-.534 to -.001) | .049 |
| Overseas | .273 (.031 to .515) | .027 | -.083 (-.308 to .142) | .467 | .305 (.063 to .547) | .013 | -.000 (-.224 to .224) | .998 |
| Disability |  |  |  |  |  |  |  |  |
| Yes | .322 (.042 to .602) | .024 | .636 (.357 to .915) | .000 | .378 (.097 to .660) | .008 | .704 (.424 to .984) | .000 |
| No | 1 |  | 1 |  | 1 |  | 1 |  |
