# Supplementary material for: Poor mental health and its impact on academic outcomes in university students before and during the COVID-19 pandemic: analysis of routine service data
Source: BJPsych Open. 2025 Mar 11;11(2):e46. doi: 10.1192/bjo.2024.868 (PMC12001929; doi:10.1192/bjo.2024.868)
Supplement: Ching et al. supplementary material 5 — Ching et al. supplementary material [file S2056472424008688sup005.docx]

Supplementary Table 5. Unadjusted and adjusted linear regression analysis on the association between potential explanatory factors and CIAO total score in the pre- and peri-pandemic samples (n = 9,517).

|  | **Unadjusted** | | | | **Fully adjusted** | | | |
| --- | --- | --- | --- | --- | --- | --- | --- | --- |
|  | Pre-pandemic | | Peri-pandemic | | Pre-pandemic | | Peri-pandemic | |
| **Fixed effects** | β/mean difference (95% CI) | p | β/mean difference (95% CI) | p | β/mean difference (95% CI) | p | β/mean difference (95% CI) | p |
| Age | .029 (.011 to .046) | .001 | -.001 (-.018 to .016) | .933 | .026 (.009 to .044) | .003 | -.003 (-.020 to .015) | .768 |
| Gender |  |  |  |  |  |  |  |  |
| Male | 1 |  | 1 |  | 1 |  | 1 |  |
| Female | -.046 (-.243 to .151) | .649 | -.146 (-.336 to .045) | .134 | -.046 (-.243 to .150) | .644 | -.146 (-.335 to .044) | .133 |
| Other | .379 (-.775 to 1.533) | .520 | .662 (-.041 to 1.364) | .065 | .298 (-.858 to 1.46) | .613 | .430 (-.277 to 1.138) | .233 |
| Sexual orientation |  |  |  |  |  |  |  |  |
| Heterosexual | 1 |  | 1 |  | 1 |  | 1 |  |
| Bisexual | .112 (-.154 to .378) | .408 | .121 (-.112 to .354) | .310 | .155 (-.112 to .421) | .255 | .104 (-.131 to .340) | .386 |
| Gay/lesbian | -.161 (-.543 to .220) | .407 | -.032 (-.403 to .338) | .864 | -.153 (-.541 to .235) | .439 | -.090 (-.464 to .283) | .635 |
| Not sure/queer | -.026 (-.322 to .270) | .865 | .122 (-.137 to .381) | .355 | -.003 (-.298 to .292) | .983 | .118 (-.142 to .378) | .375 |
| Ethnicity |  |  |  |  |  |  |  |  |
| Black | .636 (.259 to 1.013) | .001 | .722 (.384 to 1.059) | .000 | .676 (.298 to 1.054) | .000 | .824 (.487 to 1.161) | .000 |
| South Asian | .487 (.225 to .749) | .000 | .377 (.133 to .622) | .002 | .664 (.396 to .932) | .000 | .642 (.389 to .895) | .000 |
| Chinese | .431 (.101 to .761) | .010 | -.175 (-.470 to .119) | .244 | .954 (.573 to 1.335) | .000 | .414 (.072 to .756) | .018 |
| Other Asian | .618 (.259 to .976) | .001 | .522 (.189 to .855) | .002 | .900 (.527 to 1.272) | .000 | .870 (.526 to 1.215) | .000 |
| White British | 1 |  | 1 |  | 1 |  | 1 |  |
| Other White | -.067 (-.289 to .156) | .557 | -.143 (-.366 to .081) | .211 | .238 (-.012 to .488) | .062 | .198 (-.044 to .440) | .109 |
| Mixed | .124 (-.187 to .436) | .434 | .062 (-.238 to .363) | .684 | .230 (-.084 to .545) | .150 | .230 (-.072 to .532) | .135 |
| Other | .544 (.131 to .958) | .010 | .961 (.571 to 1.351) | .000 | .857 (.430 to 1.285) | .000 | 1.30 (.898 to 1.698) | .000 |
| Fee status |  |  |  |  |  |  |  |  |
| Home | 1 |  | 1 |  | 1 |  | 1 |  |
| EU | -.370 (-.591 to -.149) | .001 | -.443 (-.669 to -.218) | .000 | -.199 (-.429 to .031) | .090 | -.307 (-.540 to -.074) | .010 |
| Overseas | -.247 (-.452 to -.043) | .018 | -.493 (-.688 to -.299) | .000 | -.220 (-.425 to -.015) | .036 | -.449 (-.645 to -.253) | .000 |
| Disability |  |  |  |  |  |  |  |  |
| Yes | .718 (.482 to .953) | .000 | .856 (.615 to 1.098) | .000 | .658 (.419 to .896) | .000 | .797 (.552 to 1.042) | .000 |
| No | 1 |  | 1 |  | 1 |  | 1 |  |
