# Supplementary material for: Poor mental health and its impact on academic outcomes in university students before and during the COVID-19 pandemic: analysis of routine service data
Source: BJPsych Open. 2025 Mar 11;11(2):e46. doi: 10.1192/bjo.2024.868 (PMC12001929; doi:10.1192/bjo.2024.868)
Supplement: Ching et al. supplementary material 6 — Ching et al. supplementary material [file S2056472424008688sup006.docx]

Supplementary Table 6. Unadjusted and adjusted linear regression analysis on the association between potential explanatory factors and CORE-OM total score using imputed data (n = 9,616).

|  | **Unadjusted** | | **Fully adjusted** | |
| --- | --- | --- | --- | --- |
| **Fixed effects** | β/mean difference (95% CI) | p | β/mean difference (95% CI) | p |
| Age | -.069 (-.094 to -.044) | .000 | -.065 (-.090 to -.040) | .000 |
| Gender |  |  |  |  |
| Male | 1 |  | 1 |  |
| Female | .788 (.506 to 1.070) | .000 | .730 (.449to 1.011) | .000 |
| Other | 2.079 (.842 to 3.315) | .001 | 1.479 (.235 to 2.722) | .020 |
| Sexual orientation |  |  |  |  |
| Heterosexual | 1 |  | 1 |  |
| Bisexual | .928 (.567 to 1.290) | .000 | .819 (.456 to 1.183) | .000 |
| Gay/lesbian | -.392 (-.941 to .156) | .161 | -.126 (-.680 to .429) | .656 |
| Not sure/queer | .892 (.491 to 1.294) | .000 | .777 (.376 to 1.179) | .000 |
| Ethnicity |  |  |  |  |
| Black | 1.244 (.726 to 1.762) | .000 | 1.314 (.796 to 1.831) | .000 |
| South Asian | 1.462 (1.095 to 1.829) | .000 | 1.706 (1.329 to 2.084) | .000 |
| Chinese | 1.148 (.697 to 1.599) | .000 | 1.737 (1.215 to 2.259) | .000 |
| Other Asian | 1.698 (1.195 to 2.202) | .000 | 2.039 (1.518 to 2.560) | .000 |
| White British | 1 |  | 1 |  |
| Other White | -.237 (-.562 to .089) | .154 | .116 (-.241 to .474) | .523 |
| Mixed | .423 (-.023 to .868) | .063 | .518 (.070 to .966) | .023 |
| Other | 1.559 (.980 to 2.138) | .000 | 2.006 (1.409 to 2.602) | .000 |
| Fee status |  |  |  |  |
| Home | 1 |  | 1 |  |
| EU | -.736 (-1.062 to -.410) | .000 | -.374 (-.711 to -.038) | .029 |
| Overseas | -.077 (-.367 to .214) | .604 | -.043 (-.334 to .248) | .772 |
| Disability |  |  |  |  |
| Yes | 1.155 (.806 to 1.504) | .000 | 1.165 (.813 to 1.517) | .000 |
| No | 1 |  | 1 |  |
