# Supplementary material for: Poor mental health and its impact on academic outcomes in university students before and during the COVID-19 pandemic: analysis of routine service data
Source: BJPsych Open. 2025 Mar 11;11(2):e46. doi: 10.1192/bjo.2024.868 (PMC12001929; doi:10.1192/bjo.2024.868)
Supplement: Ching et al. supplementary material 7 — Ching et al. supplementary material [file S2056472424008688sup007.docx]

Supplementary Table 7. Unadjusted and adjusted linear regression analysis on the association between potential explanatory factors and CORE-OM risk score using imputed data (n = 9,616).

|  | **Unadjusted** | | **Fully adjusted** | |
| --- | --- | --- | --- | --- |
| **Fixed effects** | β/mean difference (95% CI) | p | β/mean difference (95% CI) | p |
| Age | -.082 (-.096 to -.068) | .000 | -.078 (-.092 to -.064) | .000 |
| Gender |  |  |  |  |
| Male | 1 |  | 1 |  |
| Female | .059 (-.100 to .217) | .470 | .018 (-.139 to .176) | .821 |
| Other | 1.460 (.763 to 2.157) | .000 | .885 (.188 to 1.582) | .013 |
| Sexual orientation |  |  |  |  |
| Heterosexual | 1 |  | 1 |  |
| Bisexual | 1.206 (1.004 to 1.409) | .000 | 1.115 (.912 to 1.318) | .000 |
| Gay/lesbian | .526 (.219 to .833) | .001 | .539 (.229 to .849) | .001 |
| Not sure/queer | .795 (.570 to 1.020) | .000 | .710 (.486 to .935) | .000 |
| Ethnicity |  |  |  |  |
| Black | .164 (-.128 to .457) | .270 | .283 (-.008 to .573) | .056 |
| South Asian | .393 (.186 to .600) | .000 | .477 (.265 to .689) | .000 |
| Chinese | .975 (.720 to 1.230) | .000 | 1.20 (.903 to 1.488) | .000 |
| Other Asian | .655 (.371 to .939) | .000 | .833 (.541 to 1.125) | .000 |
| White British | 1 |  | 1 |  |
| Other White | -.287 (-.471 to -.104) | .002 | -.144 (-.345 to .056) | .159 |
| Mixed | .206 (-.045 to .458) | .108 | .214 (-.038 to .465) | .096 |
| Other | .408 (.082 to .735) | .014 | .653 (.318 to .988) | .000 |
| Fee status |  |  |  |  |
| Home | 1 |  | 1 |  |
| EU | -.439 (-.622 to -.255) | .000 | -.304 (-.492 to -.115) | .002 |
| Overseas | .094 (-.069 to .258) | .259 | .154 (-.010 to .317) | .065 |
| Disability |  |  |  |  |
| Yes | .490 (.293 to .687) | .000 | .558 (.361 to .756) | .000 |
| No | 1 |  | 1 |  |
