# Supplementary material for: Poor mental health and its impact on academic outcomes in university students before and during the COVID-19 pandemic: analysis of routine service data
Source: BJPsych Open. 2025 Mar 11;11(2):e46. doi: 10.1192/bjo.2024.868 (PMC12001929; doi:10.1192/bjo.2024.868)
Supplement: Ching et al. supplementary material 8 — Ching et al. supplementary material [file S2056472424008688sup008.docx]

Supplementary Table 8. Unadjusted and adjusted linear regression analysis on the association between potential explanatory factors and CIAO total score using imputed data (n = 9,616).

|  | **Unadjusted** | | **Fully adjusted** | |
| --- | --- | --- | --- | --- |
| **Fixed effects** | β/mean difference (95% CI) | p | β/mean difference (95% CI) | p |
| Age | .010 (.002 to .023) | .104 | .009 (-.004 to .021) | .179 |
| Gender |  |  |  |  |
| Male | 1 |  | 1 |  |
| Female | -.079 (-.218 to .061) | .269 | -.076 (-.215 to .063) | .282 |
| Other | .669 (.578 to 1.281) | .032 | .471 (-.144 to 1.086) | .133 |
| Sexual orientation |  |  |  |  |
| Heterosexual | 1 |  | 1 |  |
| Bisexual | .154 (-.025 to .332) | .093 | .161 (-.019 to .341) | .080 |
| Gay/lesbian | -.045 (-.316 to .227) | .747 | -.061 (-.336 to .213) | .661 |
| Not sure/queer | .079 (-.120 to .278) | .436 | .088 (-.111 to .287) | .386 |
| Ethnicity |  |  |  |  |
| Black | .686 (.430 to .943) | .000 | .778 (.521 to 1.034) | .000 |
| South Asian | .365 (.183 to .547) | .000 | .609 (.421 to .796) | .000 |
| Chinese | .052 (-.171 to .276) | .646 | .658 (.400 to .917) | .000 |
| Other Asian | .572 (.323 to .822) | .000 | .918 (.660 to 1.176) | .000 |
| White British | 1 |  | 1 |  |
| Other White | -.092 (-.253 to .069) | .262 | .262 (.085 to .439) | .004 |
| Mixed | .090 (-.131 to .311) | .426 | .240 (.018 to .462) | .034 |
| Other | .602 (.315 to .890) | .000 | .957 (.662 to 1.253) | .000 |
| Fee status |  |  |  |  |
| Home | 1 |  | 1 |  |
| EU | -.426 (-.587 to -.265) | .000 | -.277 (-.443 to -.110) | .001 |
| Overseas | -.430 (-.574 to -.287) | .000 | -.396 (-.540 to -.252) | .000 |
| Disability |  |  |  |  |
| Yes | .805 (.633 to .977) | .000 | .739 (.565 to .913) | .000 |
| No | 1 |  | 1 |  |
